# Microencapsulation of *Citrus Grandis* Peel Oil Using Interfacial Precipitation Chemistry Technique for Repellent Application

**Published:** 2019

**Authors:** Norashiqin Misni, Zurainee Mohamed Nor, Rohani Ahmad

**Affiliations:** a *Department of Parasitology, Faculty of Medicine, University Malaya, Kuala Lumpur, Malaysia. *; b *Department* *of Medical Microbiology and Parasitology, Faculty of Medicine and Health Sciences, Universiti Putra Malaysia, Serdang, Selangor. *; c *Medical Entomology Unit, Institute of Medical Research, Kuala Lumpur, Malaysia.*

**Keywords:** Microencapsulation, essential oil, DEET, Citrus grandis, Aedes aegypti

## Abstract

Essential oil of Citrus family plant is known to have repellent effect against mosquito. Unfortunately, due to its high volatility effect, its repellency effect was compromised. The incorporation of essential oil in a microencapsulation formulation has been shown to help improve the stability and potency of the repellent. In this study, *Citrus grandis* peel oil (CGPO) was encapsulated by using the interfacial precipitation chemistry technique. The microencapsulated CGPO was then formulated into lotion form to produce topical repellent formulation. This study includes the characterization of microcapsules with regards to the morphology, size distribution, zeta potential, Fourier Transmission Infrared spectrophotometer (FTIR), and Thermogravity analysis (TGA). The effectiveness of the microencapsulated CGPO-lotion formulation against mosquitoes was evaluated in the laboratory setting. Results indicated that CGPO have been successfully encapsulated with 6.5 µm in diameter and zeta potential values, -47.9 mV. The FTIR analysis spectrum indicated the presence of interaction between the wall materials in microcapsules. The TGA analysis demonstrated that microencapsulation improved the thermal stability of CGPO. Repellency assay revealed that microencapsulated CGPO- based formulation possessed excellent effect compared with pure CGPO. In conclusion, CGPO was successfully encapsulated and the microencapsulation aid to improve the repellency effect of CGPO against mosquito bites.

## Introduction

N, N-diethyl-m-toluamide (DEET) is known as the most effective repellent substance to date, providing up to 8 h of protection against mosquito (1). Despite being an effective repellent, this compound has certain drawbacks. Not only it releases an unpleasant odor and causes greasy feel to the skin but is also found to cause irritation and sensitization effect (2, 3). McKinlay *et al*.*,* (4) demonstrated that high concentration of DEET gave rise to allergic or toxic reactions in some individuals making DEET formulations received lower user acceptability. As a result, the DEET-free products are finding favors with consumer and demand for compositions containing natural product are also increasing.

Nowadays, many researches on plant extract are focusing on its repellent and killing effect against blood sucking insect so that it can be used as an alternative to the existing chemical-based insecticides. Some of these plants extract such as citronella, lemongrass, pyrethrum, and geranium are commercially available and effectively used as mosquito repellent. Citronella oil is the most popular active ingredient of plant origin used in the various formulation of mosquito repellent. Some consumers, however, were unwilling to use repellent that contain citronella oil due to its strong odor and low repellency effect (5). In addition, most of the plant-based repellents already in the market are shown to have short (less than two hours) protection time (6). Therefore, there is a strong need to search for other plant extract or compound that could offer longer protection time. 

*Citrus grandis* (L). Osbeck (Pummelo) is a fruit native to the Southeast Asia and China with many nutritional and health benefits (7). Most citrus fruit is used as raw materials in conventional food processing while the peel is mostly treated as waste although the study has shown that citrus peel contains abundant phytochemicals such as flavonoid and carotenoid that might be beneficial to human health (8). As far as *C. grandis* is concerned, several studies have demonstrated that its peel oil possessed insecticidal effect against stored gran insect pests (9), mosquito larvae (10) and flies (11). Due to their great pharmaceutical and economic importance, CGPO was selected for this study.

There were few characteristics of the essential oil, which limit their effectiveness as mosquito repellents such as poor physiochemical stability, high volatility, and high thermal decomposition (12, 13). However, these characteristics may possibly be minimized by using a controlled release technique in the formulation. Microencapsulation is one of the current techniques in the controlled release formulation which has been extensively studied all over the world (14). This technique will provide the outer coating (wall) that surrounds the particle of essential oil (core) by using natural or synthetic polymer. Subsequently, it will protect the unstable core material from the environment and be able to control the volatility and extend the release rate of the essential oil (14). 

This study aims to apply the microencapsulation technique in the attempt to produce microencapsulated CGPO via interfacial precipitation chemistry. The characteristics of the microcapsules were determined by measuring their size distribution, zeta potential, Fourier Transmission Infrared spectrophotometer (FTIR) spectra, and Thermogravity analysis (TGA) curve. After the formulation into lotion form, the effectiveness of microencapsulated CGPO was evaluated against mosquito bites compared with the pure CGPO and synthetic repellent, DEET. 

## Experimental

*Materials*

Cetyl alcohol, stearic acid, vanillin, span 80, tween 80, Carboxymethyl cellulose (CMC), polyethelene glycol (PEG) 3350, Benzalkonium chloride (BKC), and diethyl-m-toluamide (DEET) were purchased from Sigma-Aldrich, USA. Dow corning 200 was purchased from Dow corning, USA. Jojoba oil, sweet almond oil, coconut oil, emulsifying wax, shear butter, and cocoa butter were purchased from BF1 Malaysia. Mosquitoes for repellent efficacy study were provided by the Institute for Medical Research of Malaysia (IMR). Susceptible strain and nulliparous three- to seven-day-old adults *Aedes aegypti* were used as test species. 

*Extraction of essential oil from plant*

Fruit peel of *C. grandis* was subjected to hydrodistillation in a clevenger-type apparatus for six hours to obtain the essential oil (15). The extracted oil was dried over anhydrous magnesium sulphate (Sigma-Aldrich, USA) in order to extract oil from the aqueous phase before storage in an airtight bottle at 4 °C for later formulation.

*Encapsulation of essential oil*

CGPO and DEET were encapsulated based on the work of Kasting *et al.* (16) and Karr *et al*. (17). The encapsulation process utilizes an interfacial precipitation chemistry to form a polysaccharide film around the dispersal droplet (18). Briefly, microcapsule walls were formed in two steps by reacting the amphiphilic macromolecule carboxymethylcellulose (CMC) with a complementary reactant benzalkonium chloride (BKC). The first step is the formation of emulsions containing droplets of the core material (CGPO or DEET) in the first wall-forming reactant solution (CMC) that preferentially accumulated at the droplet surface by polar solvent forces. The second step added the second wall-forming reactant (BKC) to the system and spontaneously precipitating the CMC to form a membrane-like wall surrounding each droplet.

For our study, the microencapsulation of GGPO and DEET involved several phases as follows: Phase A is the mixture of the active ingredient (20% CGPO or 20% DEET) with an adjuvant to form a core phase consisting 60% active ingredient and 40% adjuvant. The Dow Corning 200 (silicon oil) and vanillin were mixed and blended in a 200 mL beaker using a magnetic stirrer bar set to rotate at a minimal speed of 200 rpm at 60 °C. The solution was allowed to cool down to 45 °C before the active ingredient was subsequently added to the mixture. Stirring was continued to complete the process. In another beaker, all ingredients in phase B (cetyl alcohol, PEG 3350, SPAN 80, and TWEEN 60) were heated to melt (60 °C) and stirred to mix completely. Phase A was then slowly added in the phase B mixture and the stirring continued to form an emulsion referred to as a core emulsion mixture. 

Phase C is the aqueous solution of the first wall-forming reactant (CMC) combined with distilled water to make a 1% solution and mixed using a 40 mm diameter four-bladed propeller at 600 rpm and 45 °C. The core emulsion mixture was then dispersed into the Phase C solution at the same rotation speed and temperature. Stirring was maintained for one hour in order to produce a uniform oil-in-water dispersion (Phase E). Phase D is a mixture of the second wall-forming reactant (BKC) and distilled water. This solution was gradually added to the Phase E emulsion while slowly increasing the stirrer speed to 800 rpm in order to achieve the formation of a microcapsule wall. This mixture was then removed from heating after 120 sec and allowed to cool to ambient temperature by stirring to complete the reaction resulting in a semisolid microcapsule within the aqueous solution. Table 1 shows the amount of each composition added during encapsulation process. An additional amount of distilled water was added at the conclusion of the preparation in order to account for aqueous water losses during formulation process.

The resultant mixtures were centrifuged at 3500 rpm for 15 min. The microcapsules were washed three times with distilled water. After each wash, the samples were centrifuged and the waste water was discarded. The microcapsules were dried in the petri dish at room temperature and then dried overnight before further used. 

*Incorporation of microcapsule into lotion base*

The microencapsulated active ingredients were each formulated into a lotion base in order to produce a cosmetic repellent product that not only repelled mosquitoes but also able to moisturize skin. Emulsifying wax (2.5 g), stearic acid (5 g), shear butter (10 g), cocoa butter (5 g), coconut oil (2.5 g), sweet almond oil (10 g), and jojoba oil (5 g) were mixed together using a propeller at 400 rpm and heat at 70 °C. The mixture was then allowed to cool to 45 °C before glycerine (5 g), aloe vera gel (5 g), and a microencapsulated active ingredient (50 g) was added while continuously stirring until uniform mixture was produced. 

*Characterization of microcapsule*

*Particle size, size distribution and zeta potential*

Twenty mg samples were dispersed in 50 mL deionised water and sonicated at 25 °C for 15 minutes. The solutions were then transferred into a folded capillary cell (polycarbonate with gold plated electrodes) to test for particle size and zeta potential using Zetasizer Nano (Malvern Instrument Ltd. United Kingdom) at 25 °C. The mean particle diameter was calculated using the differential size distribution processor (SDP) intensity analysis program. The size distribution of the microcapsules was characterized statistically by polydispersity index (PDI). The lower the index value the narrower the size distribution of the microcapsules. The expected polydispersity is between 0 and 1. Values close to 0 indicate a monodispersed while values close to 1 indicate polydispersed of the particles size distribution. The measurements were done in triplicate and the average results for the measurement were recorded. 

*Morphology of the microcapsule*

Small amount of microcapsules was placed on the glass slide prior observation under optical microscope (Leica, Germany) combined with Nikon DS-Fi1 camera and NIS-Elements imaging software. 

*FTIR spectrometer analysis*

Fourier Transform Infrared spectrometer (FTIR) analysis was done to determine the chemical interaction between the two wall reagents (CMC and BKC) by using FTIR model Spectrum 400 (PerkinElmer, MA, USA). The spectra were recorded as a wave number region with expected value ranged between 400 - 4000 cm^-1 ^at 4 cm^-1 ^resolution. 

*Thermogravity analysis*

Thermal degradation of the microcapsule was carried out using themogravity analysis (TGA) model 4000 (PerkinElmer, MA, USA) to determine the decomposition temperature. About 30 mg of samples were heated from 25 to 400 °C at a heating rate of 10 °C min^-1 ^under nitrogen flow. The thermal profiles were recorded in the form of graphs and the TGA curves were derived. 

*Repellent efficacy*

Evaluation of repellent activity for each formulation followed the methods provided by the Malaysian Standard Method for repellent MS 1497:2000 (19). Bioassays were conducted using a 60 x 60 x 60 cm screened cage with two 15 cm diameter circular openings fitted with cloth sleeves. The cage had two compartments separated with a clear acrylic plastic in the middle. A fresh batch of 25 *Ae. aegypti* females were introduced into each compartment through the circular opening. Two 25 cm^2 ^areas were drawn on top of each hand of the human volunteers. One area was untreated (control); the other was pre-treated with 0.4 g of a test formulation (treatment). 

To test for any inherent repellency characteristics of the blank formulation (formulation without active ingredient), a comparison of mean landing rates per minutes of *Ae. aegypti *was conducted. Five volunteers were applied with 0.4 g of blank formulation to treatment area, leaving their second area as an untreated control. Prior to treatment, both hands were covered with rubber gloves with a 25 cm^2 ^opening corresponding to the marked area to confine mosquito bites to only the exposed areas. Both hands were exposed simultaneously in a cage for one minute and the number of mosquito lands and/or bites was recorded. The procedure was repeated 5 times with 5-min interval between each exposure.

For testing the CGPO-based lotion formulation, 0.4 g of formulation was applied evenly to the treatment area of each volunteer. 

The untreated area was used as a control. Gloves were worn to ensure the biting was focused on the exposed areas. The test began by introducing both hands into the cage containing mosquitoes for three minutes and the number of mosquito lands and/or bites was recorded. The assessment periods were one, two, four, six, and eight hours post application. The experiment was repeated with a microencapsulated DEET-based formulation as a positive control. 

The effectiveness of the formulations was based on the percent reduction of mosquito lands and/or bites on the treated arm compared with the untreated arm (control) using the following formula: 

% protection reduction = [(C-T)/C] X 100

Where* C* is the total number of mosquito lands and/or bites on the control and *T* is the total number of mosquito lands and/or bites on the treated arm. Each lotion formulation was tested on five human volunteers for three iterations.

*Data analysis*

Experiments of size distribution and zeta potential were done in triplicate and the data were presented as mean ± SD. The percentage of mosquito bites reduction data were analyzed by using SPSS Ver. 22 software. These data analyzed by split-plot ANOVA (SPANOVA) to identify mean differences between each lotion formulation and concentration over time. Prior this analysis, the data had to be transformed using the arcsine-square root transformation to better reach the parametric assumptions. A value of* P *< 0.05 was considered statistically significant. 

*Ethical approval*

This study was reviewed and approved by the Ethic Committee Faculty of Medicine of University Malaya (Ethic No: 988.11) and the safety issues for this repellent were fully considered. Individuals consented to take part of their own free will and were at freedom to withdraw from the study at any point of time without assigning any reason whatsoever. 

## Results and Discussion

*Microencapsulation of CGPO and DEET*

The suspensions of semisolid microcapsule of CGPO and DEET presented as a milky white liquid with no CGPO and DEET were observed either on the surface of the suspensions or after centrifugation of the discarded aqueous supernatant. No coalescence and/or other appearance of emulsion failure were observed, indicating very high encapsulation efficiency has taken placed (16). 

*Microcapsule characteristics*

*Morphology of Microcapsule*

**Figure 1:**
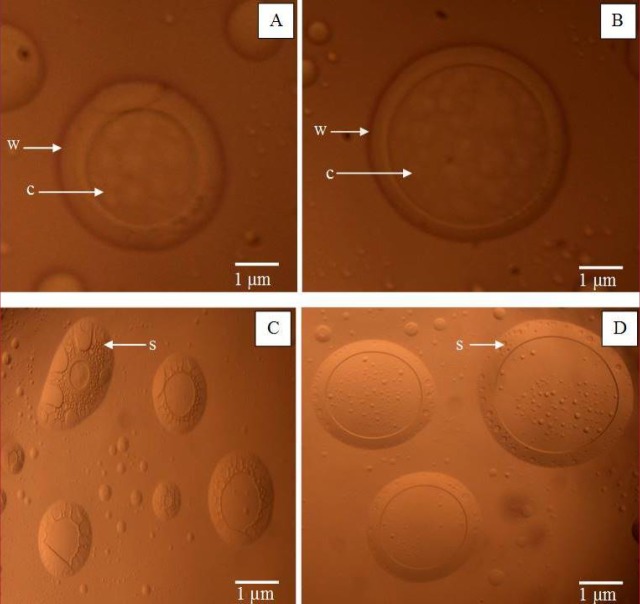
Optical micrographs of (A) single microencapsulated CGPO and B) single microencapsulated DEET (C) several microencapsulated CGPO (D) several microencapsulated DEET (10x 40 magnification). Bar represents 1 µm. w = wall, c = core and s = sponge-like surface

Figure 1 shows the optical micrograph of microcapsule CGPO and DEET. 2 (A) and 2(B) showed a single microcapsule of CGPO and DEET respectively that consisted of thick wall surrounding the core. Figure 1 also shows several microcapsules of CGPO (C) and DEET (D) that demonstrated spherical and oval shape with the surfaces of the capsules bearing rough or sponge-like structure. The microcapsules were shown distributed individually without excessive agglomeration. The diameter measurement of microcapsules in the optical micrographs indicated that diameters of the microcapsule vary in the range of 2 to 8 µm. These results were strongly supported by results of the microcapsules size distribution as described later.

*Size distributions and zeta potential*

The *Z*-average particle diameters of CGPO and DEET microcapsules indicated the size of 2.7 µm and 6.5 µm respectively, as shown in Table 2. Microencapsulation is shown to produce significantly smaller particle size of CGPO compared to DEET (*p* < 0.05) and later found to have an effect towards the particle release rate. Surface area is one of the factors known affecting the particle release rate. According to Sinko (20), the surface area increases with the decrease of particle size therefore, the release rate acts correspondingly with the surface area. Particles with smaller size (larger surface area) tend to be released faster than those having larger size. This finding corresponds with the result obtained from the repellent efficacy study as discussed later.

**Figure 2:**
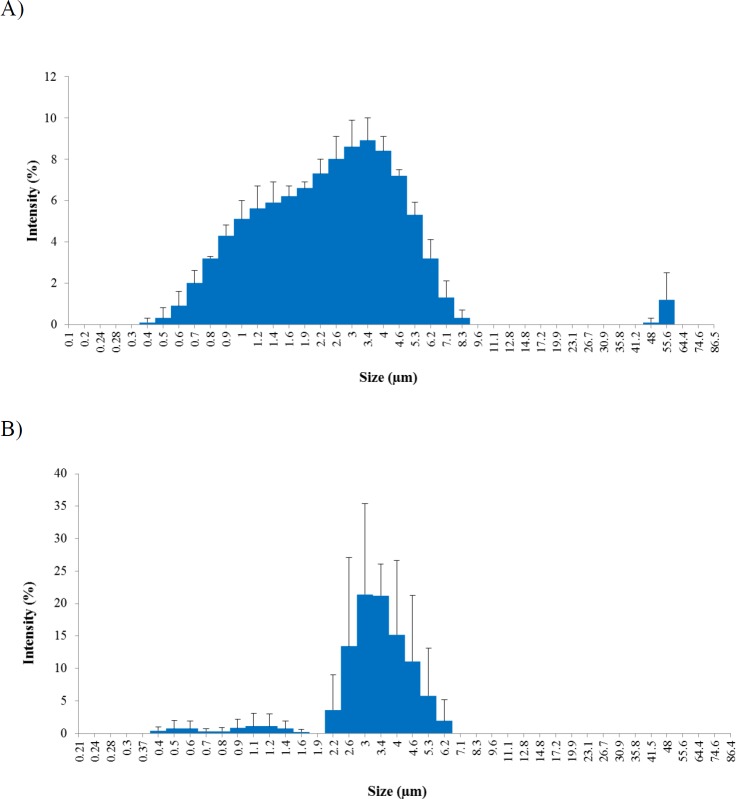
Size distributions of (A) microcapsule CGPO and (B) microcapsule DEET

The size distribution of the microcapsules was characterized by the polydispersity index (PDI). The lower the index value the narrower the size distribution of the microcapsules. The polydispersity of the microcapsules examined was found between 0 and 1. Values close to 0 indicate a monodisperse while values close to 1 indicate polydisperse in particle size distribution (21). Results obtained showed that the PDI values for both CGPO and DEET microcapsules were 0.6 and 0.4, respectively (Table 2). Thus, this indicates that DEET microcapsules were more monodisperse and had narrow size distribution compared to CGPO microcapsules. These results were confirmed by the percentage of intensity distribution histograms shown in Figure 2. CGPO microcapsule had a wider distribution (more polydisperse) ranging from 0.4 µm to 8.3 µm with PDI value closer to 1.0 (Figure 2A). In contrast, DEET microcapsule showed a narrow distribution (more monodisperse) ranging from 2.2 µm to 6.1 µm (Figure 2B).

The zeta potential is a measure of the charge on a particle surface and has been known as the indicator for the emulsion system stability. High absolute values, whether negative or positive lead to repulsive forces between particles which improve the repellency between particles, thus inhibiting aggregations (22). Low potential values on the contrary, indicated the absence of repulsive interaction resulted in agglomeration and the dispersion become unstable and precipitate (23). The zeta potential of the microencapsulated CGPO and DEET (Table 2) demonstrated strong negative charged -47.9 mV and -43.0 mV for CGPO and DEET microcapsules, respectively. These values indicate that the particles in the suspension are stable having less tendency to build up flocculation. The negative charges of microcapsules are due to the carboxylate ion (-COO^-^) and chloride ion (CI^-^) in the emulsion system. The reaction between CMC (1^st^ wall reactant) and BKC (2^nd^ wall reactant) produces two pairs of oppositely charged ions which are benzalkonium methylcellulose carboxylate and sodium chloride. Benzalkonium methylcellulose carboxylate is poorly soluble at the phase interface and precipitates to form the wall of the microcapsule while sodium chloride is typically fairly water soluble and readily dissolves in the aqueous medium (24). 

**Table 1:** CGPO and DEET microcapsules, formulation composition

**Ingredients**	**Weight (g)**
**Phase A**	
CGPO/DEET	50 g
Dow Corning 200	15 g
Vanillin	15 g
**Phase B**	
Cetyl alcohol	30 g
PEG 3350	10 g
Span 80	5 g
Tween 60	5 g
**Phase C**	
1% CMC solution	20 g
Distilled water	~ 100 g
**Phase D**	
1% BKC solution	10 g

**Table 2:** *Z *- average diameter of the microcapsules and Zeta potential

**Microcapsule**	***Z *** **- average diameter (µm ± SD)**	**Polydispersity index (PDI)**	**Zeta potential (mV ± SD)**
CGPO	2.7 ± 1.3*	0.6*	-47.9 ± 5.6*
DEET	6.5 ± 2.4	0.4	-43.0 ± 4.5

* denote as significant compared to DEET

**Figure 3:**
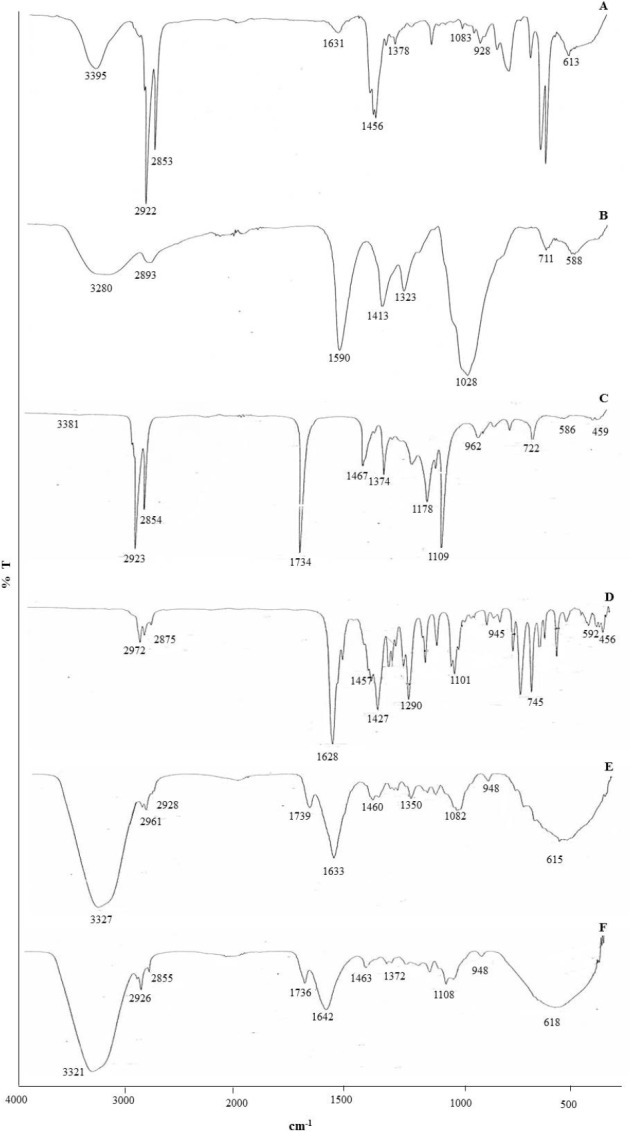
FTIR spectrums of (A) Benzalkonium chloride (BKC), (B) Carboxymethylcellulose (CMC), (C) pure CGPO, (D) pure DEET, (E) CGPO microcapsule and (F) DEET microcapsule

**Figure 4:**
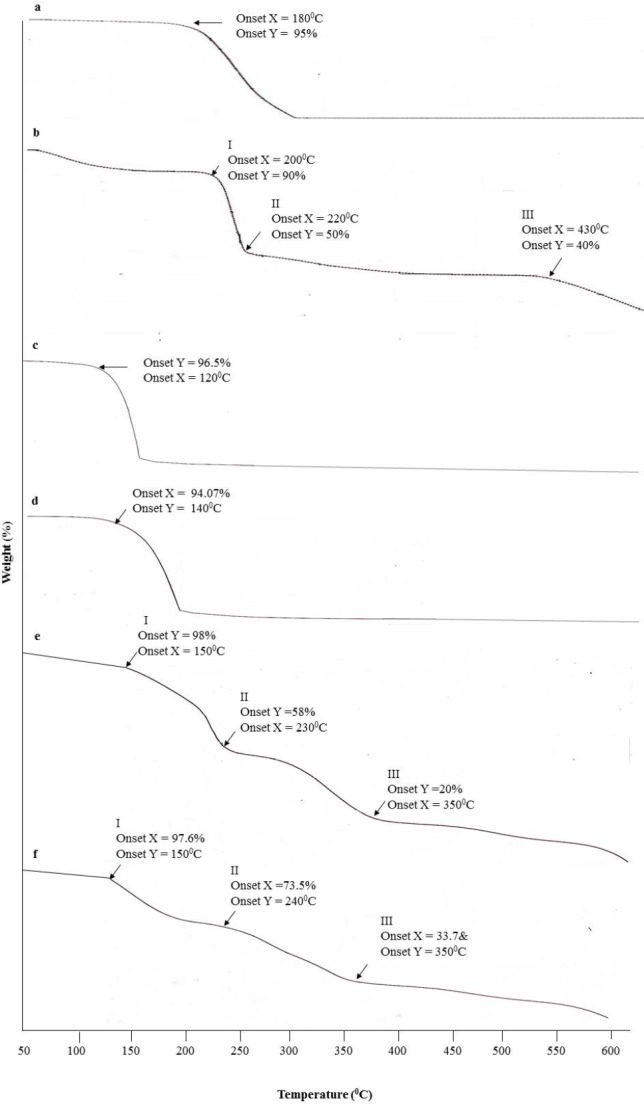
Thermal gravity analysis (TGA) of (A) Benzalkonium chloride (BKC), (B) Carboxymethylcellulose (CMC), (C) pure CGPO, (D) pure DEET, (E) CGPO microcapsule and (F) DEET microcapsule

**Figure 5:**
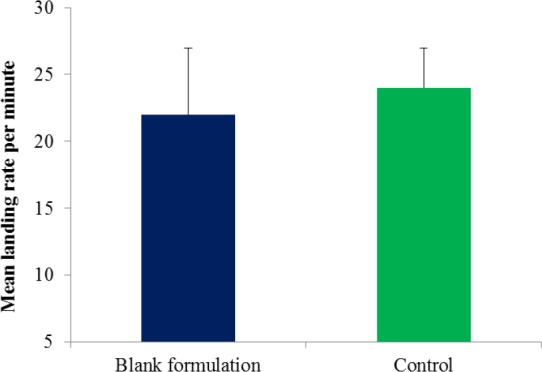
Mean landing rate of *Ae. aegypti *on area treated with blank lotion and untreated area (control). No significance was detected between blank lotion and control (*p *> 0.05). Error bars indicate SEM

**Figure 6:**
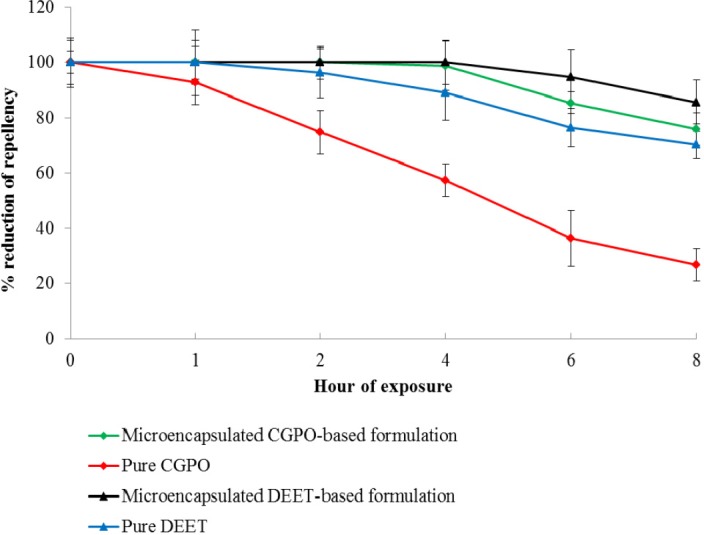
Percent repellency of microencapsulated CGPO-based lotion formulation and microencapsulated DEET-lotion based formulation compared to pure CGPO and pure DEET. Error bars indicate ± SEM.

*FTIR analysis*

FTIR analyses were employed to determine the interaction between the wall material (BKC and CMC) and the successful entrapment of the EOs in the microcapsules. The FTIR spectra of BKC, CMC, pure CGPO, microencapsulated CGPO, pure DEET, and microencapsulated DEET are shown in Figure 3. The absorption peaks of BKC (Figure 3A) show the characteristic peaks at 3395 cm^-1^ because of N-H stretching (25), while the peaks at 2922 cm^-1^, 2853 cm^-1 ^and 1456 cm^-1^ were due to the –CH stretching vibration of the surfactant tail and –CH bonding in the methyl and methylene groups, respectively (26). 

The strong and wide absorption peaks of CMC (Figure 3B) at 3280 cm^-1 ^were attributed to the stretching frequency of the O-H group (27, 28). Two typical peaks for CMC were observed at 1590 cm^-1^ and 1413 cm^-1^ due to the asymmetrical and symmetrical stretching vibration of the carboxyl (-COO^-^) group (29, 30). The peaks at 2883 and 1323 cm^-1 ^were associated with the asymmetric –CH_2_ and –OH stretching vibration, respectively. While the vibration located at 1028 cm^-1 ^was attributed to C-O stretching vibration of the cellulose backbone of the CMC (27, 31). 

The FTIR spectrum of CGPO (Figure 3C) showed typical bands related to the functional groups of the plant extracts (32, 33). The absorption peaks at 3381 cm^-1^ were due the asymmetric C-H stretching vibrations in = CH_2_. The characteristic peaks present at 2923 and 2854 cm^-1^ were relative to the vibrations of aliphatic C-H stretching vibration in –CH_3 _and CH_2_, respectively (33). The absorption peak at 1734 cm^-1^ was assigned to the C = O of saturated aliphatic ester (32) and the scissor C-H bending vibration in = CH_2 _appeared at 1467 cm^-1^ peak (33). While the symmetric C-H bending vibration in –CH_3_ was observed at 1374 cm^-1^ peak. The absorption peaks at 962 and 586 cm^-1^ in CGPO represented the C-H bending of the alkene or the aromatic groups (34). 

The FTIR analysis of DEET (Figure 3D) showed multiple absorptions peaks at 2972 cm^-1^ and 2875 cm^-1^ characterized by the aliphatic C-H stretching vibration. The absorption peak at 1628 cm^-1^ was associated with the –C = C stretching of the aromatic group. Several peaks between 1500 cm^-1^ and 1000 cm^-1 ^were assigned to the vibration of the C-C and C-H group. The vibration peaks from 945 to 456 were associated with the ‘oop’ = C-H stretch bending.

The absorption peaks at 3327 for CGPO microcapsule (Figure 3E) and 3321 cm^-1^ for DEET microcapsule (Figure 3F) indicated the presence of –OH group and N-H group from CMC and BKC, respectively (35, 36). As seen, the stretching frequency of –OH (3280 cm^-1^) in CMC and N-H group (3395 cm^-1^) in BKC shifted to 3327 cm^-1^ for CGPO microcapsule and 3321 cm^-1 ^for the DEET microcapsule. Such shifting confirmed that there is a H bonding type interaction between CMC and BKC (30, 36). The presence of new peaks at 1633 (CGPO microcapsule) and 1642 (DEET microcapsule) associated with the formation of –CONH- group which confirmed the formation of membrane around the microcapsule due to the electrostatic interaction between –COOH group of CMC and –NH_2_ group of BKC (25, 37). Most of the characteristic peaks of CGPO and DEET could be observed in the spectrum of microcapsule with minor differences in frequencies indicating the successful incorporation of essential oil or DEET into the microcapsule and chemical stability of essential oil after encapsulation. 

*Thermal analysis*

TGA curve is used to access the thermal stability and study the weight changes of samples along with temperature. The thermal analysis of BKC, CMC, pure CGPO, microencapsulated CGPO, pure DEET, and microencapsulated DEET is shown in Figure 4. The TGA curve of BKC (Figure 4A) presented one step of weight loss, began at 180 °C and completed at 300 °C. While the CMC (Figure 4B) presented the multiple weight loss steps, the first weight loss (10%) due to evaporation of water began from 50 to 200 °C, the second weight loss (50%) around 200 – 220 °C attributed to the decarboxylation process, and the third weight loss (40%) observed in the range of 220 – 430 °C was related to the main chain decomposition of the cellulose and above 430 °C the CMC started to decompose (38). 

As seen in the CGPO curve (Figure 4C), a dramatic weight loss was observed starting from 120 °C and the weight loss rate began to decrease when the temperature reached 150 °C. While for DEET (Figure 4D), the weight loss started from 140 °C and completed at 180 °C. In the case of CGPO microcapsule (Figure 4E), there was less than 2% weight loss observed up to 150 °C that may be due to the evaporation of water remaining entrapped in the capsules after the encapsulation process (37, 39). The three stages of weight loss were observed as the temperature was increased about 150 °C. After 150 °C until 230 °C, the essential oil reached the boiling point and the microcapsule wall broke. Part of the core material was released from the microcapsule resulted in a 58% weight loss. Compared with essential oil, the microcapsule had slower weight loss rate under temperature range (40). The second weight loss stage (20%) ranging from 230 °C and 350 °C was due to the degradation of microcapsule wall and the temperature range was consistent with that of the third weight loss stage and complete degradation for the CMC and BKC, respectively (40). The third stage of weight loss observed above 350 °C was due to the residual degradation of the microcapsule wall (37). These respective weight losses were also observed in the case of DEET microcapsule (Figure 4F) with minimal difference in the temperature and weight loss.

From these results, it can be concluded that the encapsulation significantly improved the thermal stability of active ingredients (CGPO and DEET), suggesting that the wall materials (CMC and BKC) encapsulated these compounds appropriately (37, 40). In addition, due to the compact encapsulation of the DEET and CGPO, the thermal stability curve of the microcapsule looked more complicated than their pure compounds thus suggesting that the DEET and CGPO were most likely being successfully encapsulated (39).

*Efficacy studies*

Statistically, no significant difference was detected between the mean landing rate per minute of *Ae. aegypti* on the blank formulation area and untreated area (Figure 5). This indicated that the blank lotion did not possess inherent repellent properties and therefore did not affect the repellency response of the CGPO and DEET. Figure 6 shows the percentage reduction of repellent efficacy for the microencapsulated CGPO-based lotion formulation and microencapsulated DEET- based lotion formulation against *Ae. aegypti *for six hours of exposure. The results obtained indicated that the microencapsulated CGPO-based lotion formulation provides complete protection (100%) for 2 h and then followed by high protection (98.82%) for the next 4 h of exposure. After 8 h of exposure, the protection was eventually reduced to 75.86% against *Ae. aegypti*. As for the microencapsulated DEET-based lotion formulation, results showed that complete protection (100%) was maintained for 4 h after that > 80% of protection was sustained up to 8 h post application.

Statistical analysis revealed that the microencapsulated CGPO-based lotion formulation showed no significant difference compared with the microencapsulated DEET-based lotion formulation at 4 h of exposure (*p* > 0.05). Although the microencapsulated CGPO-based formulation possessed similar efficacy as the microencapsulated DEET up to 4 h post application, it was unable to sustain the high protection as long as microencapsulated DEET-based lotion formulation (8 h). As discussed earlier, the microcapsule diameter size plays an important role in the microcapsule release rate. Microcapsule with smaller size tends to have high release rate due to the large surface area and microcapsule with the high release rate providing the short duration of protection. This could be the possible explanation to why the microencapsulated CGPO-based lotion formulation only possessed > 75% protection after 8 h post application. The attempt to perform a similar test on 20% concentration of pure CGPO and pure DEET found the absence of complete protection for CGPO and only 2 h of protection for DEET. This finding suggested that the encapsulation of CGPO or DEET has helped these compounds to improve the repellency duration against mosquito bites.

## Conclusion

The results obtained from this study indicate that CGPO and DEET were successfully encapsulated using the interfacial precipitation chemistry technique. The particle sizes of the microencapsulated CGPO and DEET were 2.7 and 6.5 µm, respectively. Zeta potential analysis indicated the stability of microencapsulated CGPO and DEET in the emulsion system. The FTIR spectra indicated the presence of interaction between the CMC and BKC in microcapsule; thus suggesting the successful encapsulation of active ingredients into the wall materials. The thermal analysis suggested that the core materials were properly encapsulated within the wall materials providing good thermal stability for the core materials. The formulation of microencapsulated CGPO in the lotion form demonstrated a remarkable potency as repellent against mosquito bites by providing outstanding repellency effect compared to pure compounds besides showing comparable effects compared with the gold standard for repellent, DEET. In conclusion, by employing the microencapsulation technique in the plant-based repellent product development particularly the volatile essential oil helps to improve their stability as well as their effectiveness as mosquito repellent. 
